# Coating of Conducting and Insulating Threads with Porous MOF Particles through Langmuir-Blodgett Technique

**DOI:** 10.3390/nano11010160

**Published:** 2021-01-10

**Authors:** Sakandar Rauf, Miguel A. Andrés, Olivier Roubeau, Ignacio Gascón, Christian Serre, Mohamed Eddaoudi, Khaled N. Salama

**Affiliations:** 1 Sensors Lab, Advanced Membranes & Porous Materials Centre (AMPMC), Computer, Electrical, and Mathematical Sciences and Engineering (CEMSE) Division, King Abdullah University of Science and Technology (KAUST), Thuwal 23955-6900, Saudi Arabia; sakandar.rauf@kaust.edu.sa; 2 Instituto de Nanociencia y Materiales de Aragón (INMA), CSIC and Universidad de Zaragoza, 50009 Zaragoza, Spain; mandres@unizar.es (M.A.A.); roubeau@unizar.es (O.R.); 3 Departamento de Química Física, Universidad de Zaragoza, 50009 Zaragoza, Spain; 4 Institut des Matériaux Poreux de Paris, FRE 2000 CNRS Ecole Normale Supérieure de Paris, Ecole Supérieure de Physique et de Chimie Industrielles de Paris, PSL Research University, 75005 Paris, France; christian.serre@ens.psl.eu; 5 Functional Materials Design, Discovery & Development Research Group (FMD3), Advanced Membranes & Porous Materials Center, Division of Physical Sciences and Engineering, King Abdullah University of Science and Technology (KAUST), Thuwal 23955-6900, Saudi Arabia; mohamed.eddaoudi@kaust.edu.sa

**Keywords:** metal-organic framework (MOF), MIL-96(Al), Langmuir-Blodgett (LB) films, fiber, thread, conductive thread, thin films, textile coatings, functional textiles

## Abstract

The Langmuir-Blodgett (LB) method is a well-known deposition technique for the fabrication of ordered monolayer and multilayer thin films of nanomaterials onto different substrates that plays a critical role in the development of functional devices for various applications. This paper describes detailed studies about the best coating configuration for nanoparticles of a porous metal-organic framework (MOF) onto both insulating or conductive threads and nylon fiber. We design and fabricate customized polymethylmethacrylate sheets (PMMA) holders to deposit MOF layers onto the threads or fiber using the LB technique. Two different orientations, namely, horizontal and vertical, are used to deposit MIL-96(Al) monolayer films onto five different types of threads and nylon fiber. These studies show that LB film formation strongly depends on deposition orientation and the type of threads or fiber. Among all the samples tested, cotton thread and nylon fiber with vertical deposition show more homogenous monolayer coverage. In the case of conductive threads, the MOF particles tend to aggregate between the conductive thread’s fibers instead of forming a continuous monolayer coating. Our results show a significant contribution in terms of MOF monolayer deposition onto single fiber and threads that will contribute to the fabrication of single fiber or thread-based devices in the future.

## 1. Introduction

The interest of the scientific community in the preparation, characterization, and study of metal-organic frameworks (MOFs) has continuously grown during the last forty years [1,2,3]. MOFs possess exceptional chemical versatility and tunable porosity that make them promising materials for a wide range of applications [4], including, among others, separation science [5,6,7,8], health [9,10,11], and the environment [12]. The development of many MOF applications nevertheless requires the formation of uniform MOF thin films onto appropriate surfaces [13,14,15,16]. In particular, the deposition of MOF thin coatings onto the surface of textiles is a challenging target that has been scarcely explored. Previous studies have shown that it is possible to integrate MOF and fabrics and the use of mixed materials in a variety of applications, such as detoxifying filters [17], protection against solar radiation [18,19], and gas sensing [20]. However, in general, complicated procedures that require more than two steps need to be used to obtain the desired MOF/textile combinations: fibroin processing during several days, followed by electrospinning and curing [17]; fiber incubation at controlled temperature into different solutions during several hours followed by drying and washing [18]; reactants grinding followed by hot-pressing, cleaning, and drying [19]; fiber immersion into the reaction mixture, sonication, heating at a controlled temperature, washing, and drying [20]. Moreover, following these procedures, MOFs are not located specifically on the surface of the fibers, but also inside them, which is not always convenient for some applications and generally requires the use of large MOF amounts. 

In this context, the use of the Langmuir-Blodgett (LB) technique [21,22] for MOF nanoparticle (NP) deposition presents some significant advantages since homogeneous and dense MOF monolayers can be deposited on the surface of different materials without any pre-treatment of the substrate, using only a minimal MOF amount. Previous studies in the literature have dealt with the deposition of colloidal NPs and metallic NPs onto different substrates using the LB technique [23,24,25]. Recently, we have shown the fabrication of monolayer films of NPs of MIL-101(Cr) [26,27] and MIL-96(Al) [28,29,30], with sizes of ca. 50 and 200 nm respectively, onto substrates of different nature, including interdigitated textile electrodes made of a silver-coated conductive thread onto different fabrics, and their use as a textile humidity sensor [30]. In this contribution, we present a systematic study about the direct fabrication of monolayers of MIL-96(Al) NPs onto different commercially available fibers and threads to explore what kind of textile fabrics could be more suitable for the development of MOF-coated textiles. We demonstrate that it is possible to deposit MOF LB films onto different types of fibers or threads, without the need for a more or less elaborated pre-treatment that would modify the fiber or thread surface or could imply expensive equipment (e.g., plasma treatment).

## 2. Materials and Methods 

**Figure 1:**
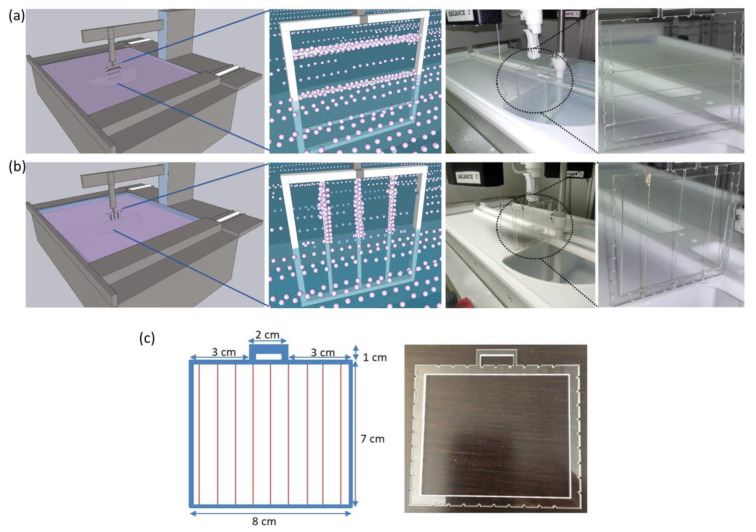
Schematic depiction and photograph of the setup used for Langmuir-Blodgett (LB) deposition in horizontal (**a**) and vertical (**b**) configurations of the fiber or threads. (**c**) Dimensions and picture of the holder showing vertical and horizontal grooves for fixing the fiber or threads.

MIL-96(Al) nanoparticles of size ca. 200 nm were obtained following a procedure previously reported [31]. Details on NPs characterization have already been reported by some of us [28,29,31]. Five different conductive and non-conductive threads and a nylon fiber were used in these studies to test the deposition of MIL-96(Al) NPs. The non-conductive fiber and threads used were cotton thread (white color, no brand), nylon fiber (invisible thread, 100% nylon from Hemline), and dental floss thread (dental floss, Orex). Moreover, cotton thread treated with acetic acid was also used in our experiments, following the treatment protocol reported by Owyeung et al. [32]. Film deposition on conductive threads was performed on Kookye silver conductive thread (Kookye conductive thread, resistance 2 Ω/ft, Kookye^®^ from Pinetree Electronics Ltd., Canada), Liberator 40 Vectran silver-coated conductive thread (Liberator 40^®^, resistance 1 Ω/ft, Syscom Advanced Materials Inc., USA), and BCP conductive sewing thread (Brand BCP, no resistance information). All of these threads were purchased from Amazon USA and used as received. All the samples used in these studies are made out of many single fibers twisted or bundled together to make a thread except nylon fiber, as this is a single fiber. Therefore, we used the terminology nylon fiber, and for all other samples, we used the word thread at the end of each material name. The custom-built square holders of transparent polymethylmethacrylate sheets (PMMA) (Figure 1) were fabricated using a CO_2_ laser (VLS 3.5 Desktop laser platform). These PMMA holders were used to hold the fiber or threads during the LB film deposition process. 

LB films were fabricated using a commercially available KSV-NIMA trough, model 2000-System 3, with dimensions 775 × 120 mm and a symmetrical double-barrier system. Surface pressure was registered by means of an electrobalance using the Wilhelmy plate method. Previous studies have shown that dense and homogeneous MIL-96(Al) monolayers can be formed at the air-water interface following this procedure (see more details in previous publications) [28,29,30]: after trough and water surface cleaning, 8 mL of a MOF dispersion in chloroform (0.2 mg of MOF per mL) prepared through probe ultrasonication were spread drop by drop using a Hamilton microsyringe. Then, after solvent evaporation, Langmuir films were compressed at 6 cm^2^/min to achieve the desired transfer pressure (30 mN/m).

LB films were deposited onto samples using the custom made PMMA holders. Each holder incorporated different types of fiber or threads up to 3 samples (Figure 1). These holders were initially immersed in the water and were withdrawn at 1 mm/min after reaching the target surface pressure (30 mN/m). All samples were allowed to dry in the air overnight, and then they were stored in a desiccator until characterization was performed. MOF deposition onto the fibers was characterized using two field emission scanning electron microscope (FE-SEM) instruments: an FEI Quanta 3D FEG and an Inspect F50 system, both operated with an accelerating voltage of 10 kV. A coating of 5 nm thickness of Ir or Pt was applied to the samples before SEM inspection. At least three different samples of each thread or fiber and deposition orientation were analyzed. Coverage analysis was performed using ImageJ software.

## 3. Results and Discussion

To allow an effective deposition of MOF particles onto the fiber or threads using the LB method, the sample needs to be kept elongated during the film transfer. For this, the first essential step was designing a custom holder, shown in Figure 1. MOF monolayer films of 200 nm MIL-96(Al) particles with a homogeneous size were then deposited onto nylon fiber (insulating) and five different types of threads, namely two insulating (cotton thread and dental floss thread) and three conductive threads (Kookye conductive thread, Liberator 40 conductive thread and BCP conductive thread), all at 30 mN/m using the LB method (see experimental section). Two deposition orientations, namely, horizontal and vertical deposition, were used for the LB coating of MIL-96(Al) NPs onto the nylon fiber and five other threads. In the case of horizontal deposition, the fiber or threads were mounted onto the frame in the horizontal direction, and in the case of vertical deposition, the nylon fiber or threads were placed in a vertical direction in reference to the water surface where the MOF dense monolayer was previously formed. The use of custom made holders allows the coverage of multiple samples in a single run, which is also an exciting feature of our method in terms of getting higher output in one deposition cycle. LB films deposited onto the samples were characterized using scanning electron microscopy (SEM).

**Figure 2:**
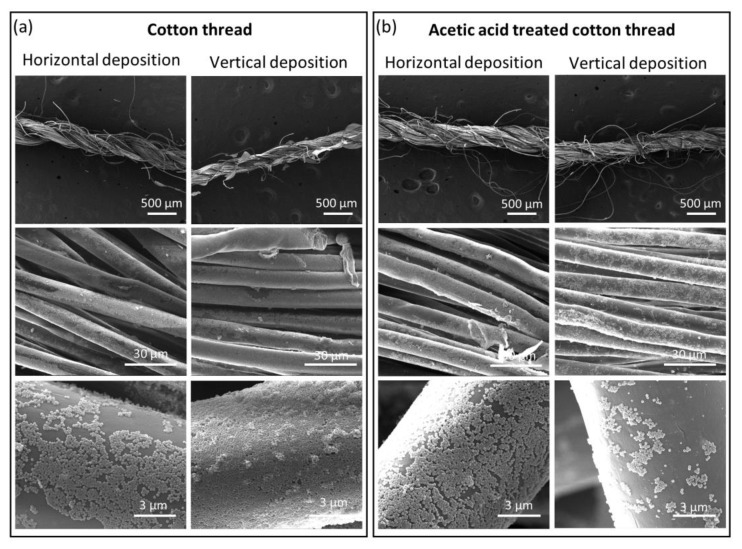
Representative SEM images for horizontal and vertical deposition of metal-organic framework (MOF) nanoparticles (NPs) onto pristine cotton thread (left, **a**) and cotton thread treated with acetic acid (right, **b**). From top to bottom, each column shows the increased magnification of the threads.

In the case of non-conducting samples, single nylon fiber and two threads, namely, cotton thread and dental floss thread, were used for LB film deposition. In the case of cotton thread, the deposition was performed on both pristine threads and threads treated with acetic acid (Figure 2). The latter is a common procedure for chemical purification of cellulose [33,34], either in crude form or processed as fibers. The effect of acetic acid is the hydrolysis of the non-crystalline regions of the cellulose [35], and we expected a possible increase in the interaction between the cotton thread and the MOF particles. We followed the protocol optimized by Owyeung et al. where they used commercial cotton thread like in our case, and they reported that acetic acid treatment increased the interaction of a dye with the cotton thread by removing any wax or non-cellulosic coating onto the fibers [32]. Figure 2 depicts the resulting coatings, as observed through SEM. Additional SEM images can be found in Supplementary Material, Figure S1. After the acetic acid treatment, a slight increase in the diameter of the cotton thread was observed (from 352 ± 41 µm to 392 ± 50 µm, Table S1) which is due to the partial opening of the thread fibers, and these results are in agreement with the previously reported studies about the cotton thread modification with acetic acid [32].

It can be seen that for the pristine cotton thread, vertical deposition orientation gives the best monolayer coverage of MIL-96(Al) (70–99%), the thread being only partially coated in the case of horizontal deposition (60–80%). However, even for the more favorable vertical configuration, full coverage of the threads was not observed. On the contrary, in the case of acetic acid-treated cotton thread, the horizontal deposition gives much better results than the vertical deposition (89–94% vs. 30–45%). This difference may be ascribed to the different interactions underlying each transfer type. While horizontal deposition simply occurs by the removal of material from the air-water interface during thread withdrawal from inside the water, vertical deposition implies the formation of a concave meniscus. The acetic acid treatment partially opens the thread fibers increasing the inhomogeneity on the surface of the thread and the tortuosity of individual fibers. This inhomogeneity probably disrupts the film in the meniscus zone leading to a poorer interaction and coverage. The observed coating for horizontal deposition is also significantly better than on pristine cotton thread, with a more homogenous and complete coverage of the thread. This can reasonably be ascribed to the removal of species potentially present on the surface of the cotton thread through the acetic acid pre-treatment. Therefore not only the surface roughness but also the physical and chemical homogeneity play a key role in the deposition of LB films of NPs. 

**Figure 3:**
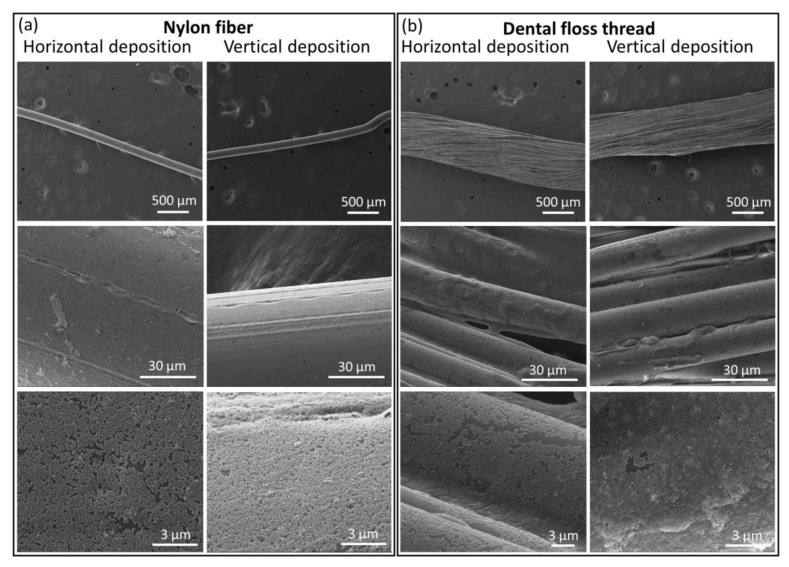
Representative SEM images for horizontal and vertical deposition of MOF NPs onto nylon fiber (left, **a**) and dental floss thread (right, **b**). From top to bottom, each column shows increased magnification.

The deposition of MIL-96(Al) NPs onto the nylon fiber and dental floss thread is depicted in Figure 3. Additional SEM images are included in Supplementary Material, Figure S2. We used a commercially available dental floss thread, consisting of a bundle of nylon fibers winded together, having a wax coating. Overall, a good coating of the fibers is obtained in all cases, for both nylon fiber and dental floss thread, and using either horizontal or vertical depositions. The highest surface coverage is obtained on nylon fiber using vertical deposition (84–97%), with a denser and more homogeneous coating than horizontal deposition (80–96%). This high coverage is achieved without the need for any special pre-treatment, such as the plasma treatment of PMMA optical fibers prior to silica NPs deposition reported by Kohoutek et al. [25]. Moreover, the nylon fiber diameter (126 ± 8.3 µm, Table S1, Supplementary Material) is significantly smaller than the value reported for those PMMA optical fibers (ca. 3 mm). Concerning the homogeneity of the MOF deposits, almost no aggregates are observed in nylon fiber samples. SEM images in Supplementary Material, Figure S3 illustrate this monolayer character of the MOF coating. On the other hand, some uncovered areas, as well as some regions with more than one layer of MOF NPs, can be observed in the case of dental floss thread, the latter especially when the vertical deposition was used. Therefore although a good coating can be obtained on dental floss thread (66–94% and 70–75% for vertical and horizontal transfer), greater extents of deposition are achievable on single nylon fibers. This could be ascribed to either or both the bundled nature and the wax coating of dental floss thread. 

Thus, among the non-conductive samples, both cotton thread and nylon fiber with vertical deposition gave similar results in terms of homogeneity and film quality, with nylon fiber performing the best in coverage degree. The fact that acetic acid treatment of cotton thread results in an improved coating, while that on nylon fiber is better than on dental floss thread in which a wax surface coating is present, may be associated with the necessity to clean the surface of the thread. To summarize these results, Table S1 in Supplementary Material includes all the coverage values for the non-conductive fiber and threads deduced from the analysis of SEM images.

**Figure 4:**
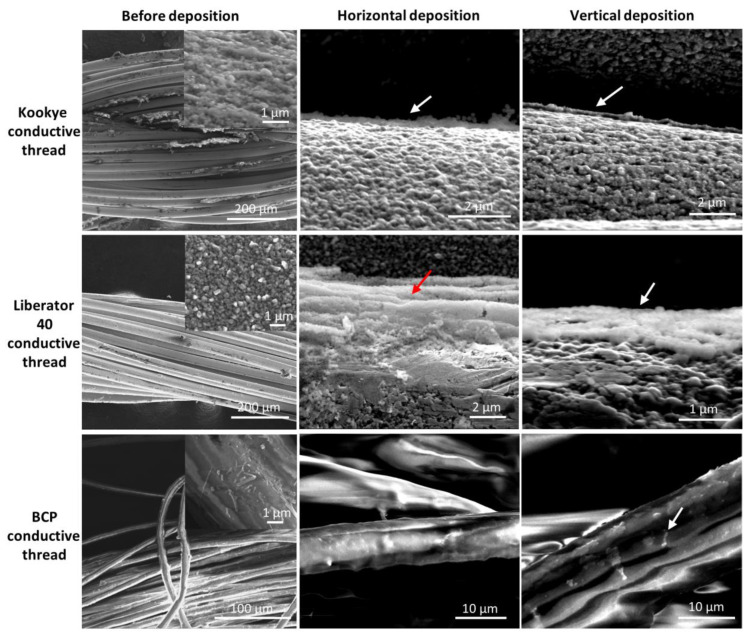
SEM images of the three conductive threads studied, as indicated, prior to deposition (**left**), after LB deposition of MOF NPs in horizontal (**middle**) and vertical (**right**) configurations. The red and white arrows highlight the MOF NPs deposits.

To further evaluate the application of our coating strategy, we coated MIL-96(Al) onto three different commercially available conductive threads with different diameters (Table S2, Supplementary Material), again using horizontal and vertical deposition orientations. Figure 4 shows the SEM micrographs of the conductive threads before and after the deposition of the MOF NPs layer. Additional SEM images are included in Supplementary Material, Figure S4. It is important to note that these conductive threads already have a coating of metal nanoparticles (Ag), resulting in a rough surface. In some cases, it was not easy to clearly distinguish MOF NPs from the conductive coating. For that purpose, back-scattered electron detector (BSE) proved to be useful (Supplementary Material, Figure S5), where MOF coated areas appeared as darker zones in the images due to the smaller atomic number of the metallic center (Al, Z = 13) in comparison to the Ag NPs (Z = 47).

The BCP conductive thread showed the poorest deposition of MOF particles, with only a few small areas covered in the horizontal and vertical deposition. Moreover, resistance showed an increase compared to the uncoated thread (from 500 Ω to 2000 Ω), which can be reasoned by a partial loss of the conductive coating during Langmuir film fabrication. Nonetheless, the conductivity of Kookye and Liberator 40 conductive threads remained unchanged after MOF LB deposition (3.0 Ω for Kookye conductive thread and 0.5 Ω for conductive thread), which demonstrates the potential applications of LB films directly deposited onto conductive threads for application in chemical sensors (e.g., as interdigitated electrodes in textile sensors [30]). The Kookye conductive thread also showed a rather poor MOF particle deposition in both deposition orientations, although much better than the BCP conductive thread. Indeed, areas covered with a continuous film of MOF particles can be observed but separated by large areas with no coating present. A more efficient deposition of MOF NPs was obtained in the case of Liberator 40 conductive thread, the films being more continuous and homogeneous in height in the vertical configuration as compared to the horizontal deposition. However, even in this more favorable case, the deposition does not occur as a monolayer, as obtained in the case of non-conductive cotton thread and nylon fiber. The red arrow in Figure 4 highlights that the MOF particles deposit preferentially at the fiber junctions or the grooves between the small conductive fibers inside the fiber bundle of the conductive thread. Contrary to the insulating fiber and threads, deposition on the conductive threads results in a multilayer coating with the MOF particles. The initial roughness of the conductive threads is one of the most likely parameters at the origin of this. The bundle nature of the conductive threads also plays a role in their relatively poor coating, but since a reasonable coating was obtained for the bundle of nylon fibers in dental floss thread, this is likely only comparatively less relevant. 

## 4. Conclusions

This work reports the direct deposition of MOF LB films onto conventional and conductive threads with diameters below 1 mm and of different nature, also using different orientations in the deposition process. Overall, the deposition process we report does not require any special pre-treatment of the samples and is reproducible, either for the nylon fiber or various threads of each deposition batch or from deposition batch to deposition batch. It has been demonstrated that the chemical nature, surface roughness, and type of fiber or thread, either single-fiber or bundles, as well as deposition orientation all appear to influence the ability to obtain a monolayer coating of MOF particles. Moreover, in the case of conductive threads, the nature of both fiber and conductive coating plays a key role in LB deposition to avoid the leaching of the metallic NPs by immersion into the water subphase. The deposition of small amounts of MOF NPs using the LB technique did not alter the thread conductivity, which is essential for implementing these threads into electronic devices. Moreover, the MOF films were not removed by bending the nylon fiber or threads. Further studies of these MOF LB coated fibers will pave the way for the design of single-fiber or thread-based devices that could be integrated into chemical or gas sensors. Particularly, abrasion resistance and immersion stability, among other properties, would have to be addressed for the design of these devices.

## Data Availability

Data is contained within the article or supplementary material.

## Supplementary Materials

The following are available online at https://www.mdpi.com/2079-4991/11/1/160/s1, Figure S1 to Figure S5: Additional SEM images for horizontal and vertical deposition, Table S1: Diameter of nylon fiber, cotton thread and dental floss thread and coverage (in %) obtained from SEM images, Table S2: Diameter of conductive threads.
