# Supplementary material for: Coating of Conducting and Insulating Threads with Porous MOF Particles through Langmuir-Blodgett Technique
Source: Nanomaterials (Basel). 2021 Jan 10;11(1):160. doi: 10.3390/nano11010160 (PMC7828012; doi:10.3390/nano11010160)
Supplement: Supplementary file 1 [file nanomaterials-11-00160-s001.pdf]

nanomaterials

Supplementary Material

Coating of Conducting and Insulating Threads with Porous MOF Particles through Langmuir-Blodgett Technique

Sakandar Rauf [1,†], Miguel A. Andrés [2,3,†], Olivier Roubeau [2], Ignacio Gascón [2,3,*], Christian Serre [4], Mohamed Eddaoudi [5] and Khaled N. Salama [1,*]

1 Sensors Lab, Advanced Membranes & Porous Materials Centre (AMPMC), Computer, Electrical, and Mathematical Sciences and Engineering (CEMSE) Division, King Abdullah University of Science and Technology (KAUST), Thuwal 23955-6900, Saudi Arabia; sakandar.rauf@kaust.edu.sa
2 Instituto de Nanociencia y Materiales de Aragón (INMA), CSIC and Universidad de Zaragoza, 50009 Zaragoza, Spain; mandres@unizar.es (M.A.A.); roubeau@unizar.es (O.R.)
3 Departamento de Química Física, Universidad de Zaragoza, 50009 Zaragoza, Spain
4 Institut des Matériaux Poreux de Paris, FRE 2000 CNRS Ecole Normale Supérieure de Paris, Ecole Supérieure de Physique et de Chimie Industrielles de Paris, PSL Research University, 75005 Paris, France; christian.serre@ens.psl.eu
5 Functional Materials Design, Discovery & Development Research Group (FMD3), Advanced Membranes & Porous Materials Center, Division of Physical Sciences and Engineering, King Abdullah University of Science and Technology (KAUST), Thuwal 23955-6900, Saudi Arabia; mohamed.eddaoudi@kaust.edu.sa
* Correspondence: igascon@unizar.es (I.G.); khaled.salama@kaust.edu.sa (K.N.S.)
† These authors contributed equally.

Table S1. Coverage (in %) obtained from SEM images for non-conductive cotton thread, nylon fiber and dental floss thread coated with MOF LB films either in the horizontal or vertical direction. At least four different images and different zones were analyzed for each class.

Sample Type	Diameter (µm)	Transfer direction	Minimum coverage (%)	Maximum coverage (%)
**Cotton thread**	352 ± 41	Vertical	70.4	99.2
		Horizontal	60.5	79.7
**Cotton thread (treated)**	392 ± 50	Vertical	29.9	44.6
		Horizontal	88.6	93.7
**Nylon fiber**	126 ± 8.3	Vertical	84.1	96.9
		Horizontal	80.7	96.1
**Dental floss thread**	717 ± 43	Vertical	65.8	93.7
		Horizontal	70.0	75.4

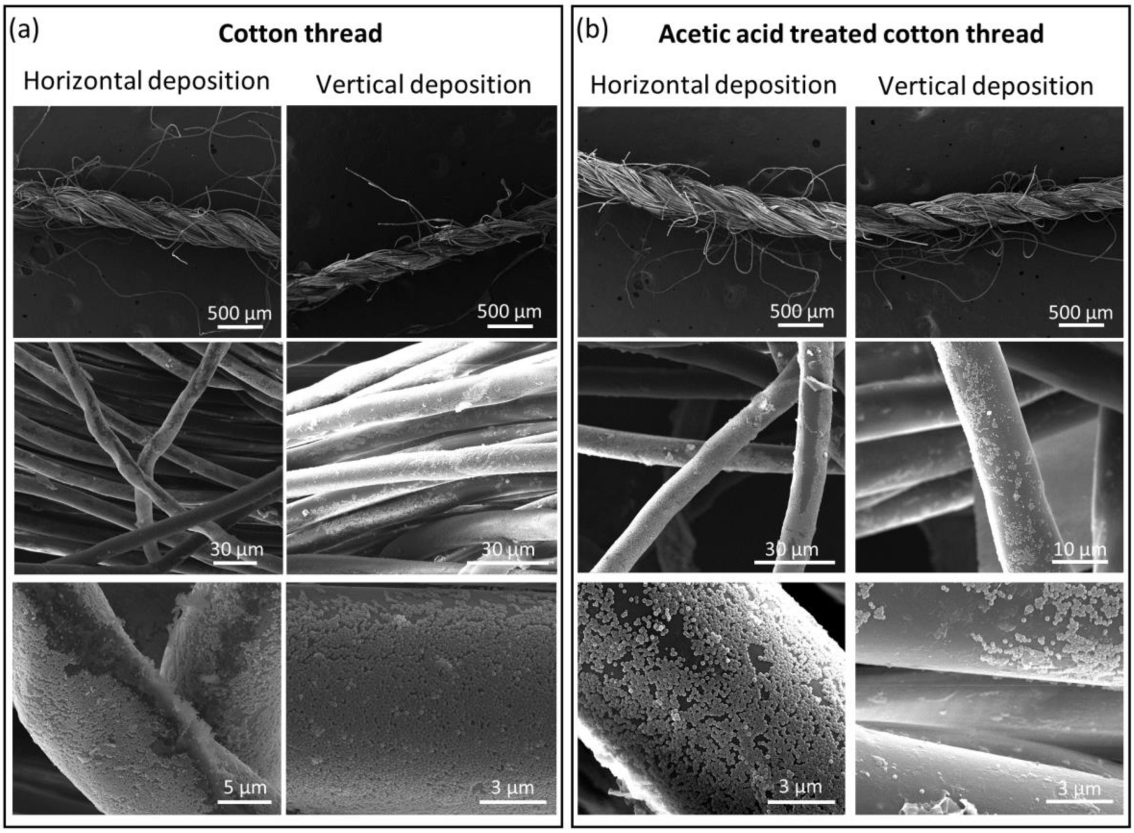

Figure S1. Additional SEM images for horizontal and vertical deposition of MOF NPs onto pristine cotton thread (left, **a**) and cotton thread treated with acetic acid (right, **b**). From top to bottom, each column shows the increased magnification of the fibers.

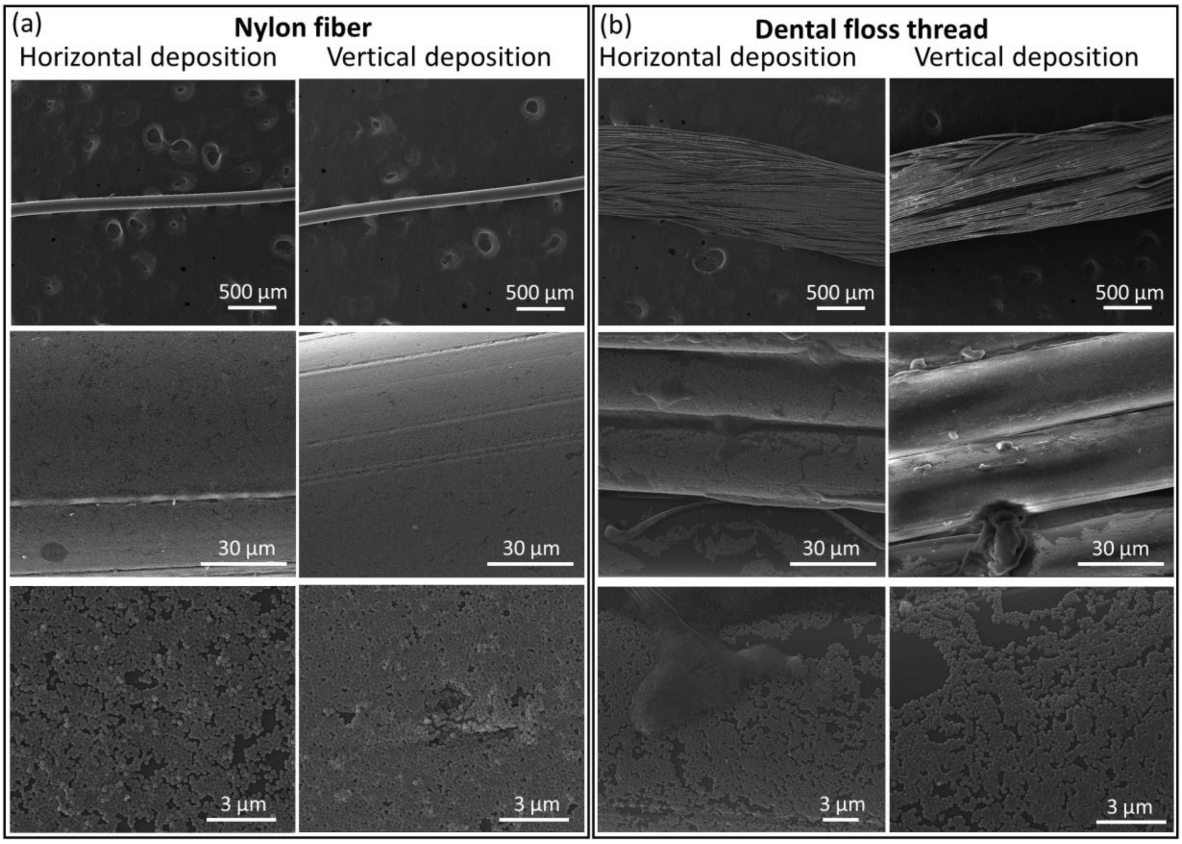

Figure S2. Additional SEM images for horizontal and vertical deposition of MOF NPs onto nylon fiber (left, **a**) and dental floss thread (right, **b**). From top to bottom, each column shows increased magnification.

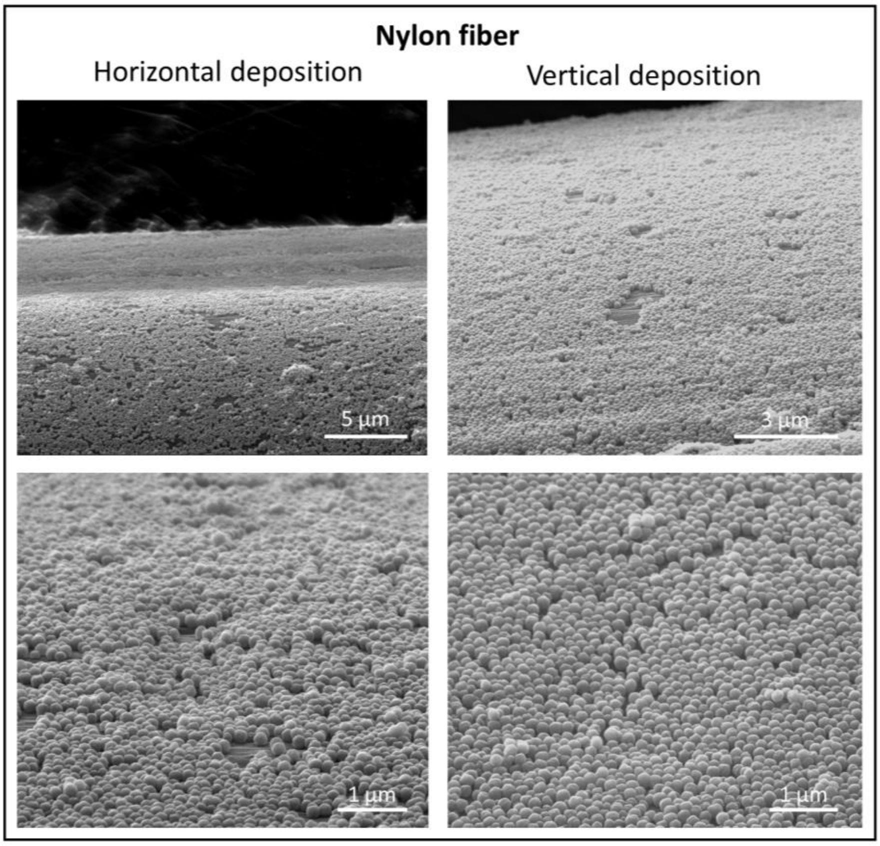

Figure S3. SEM images showing details of the monolayer coverage of MOF film in nylon fiber samples.

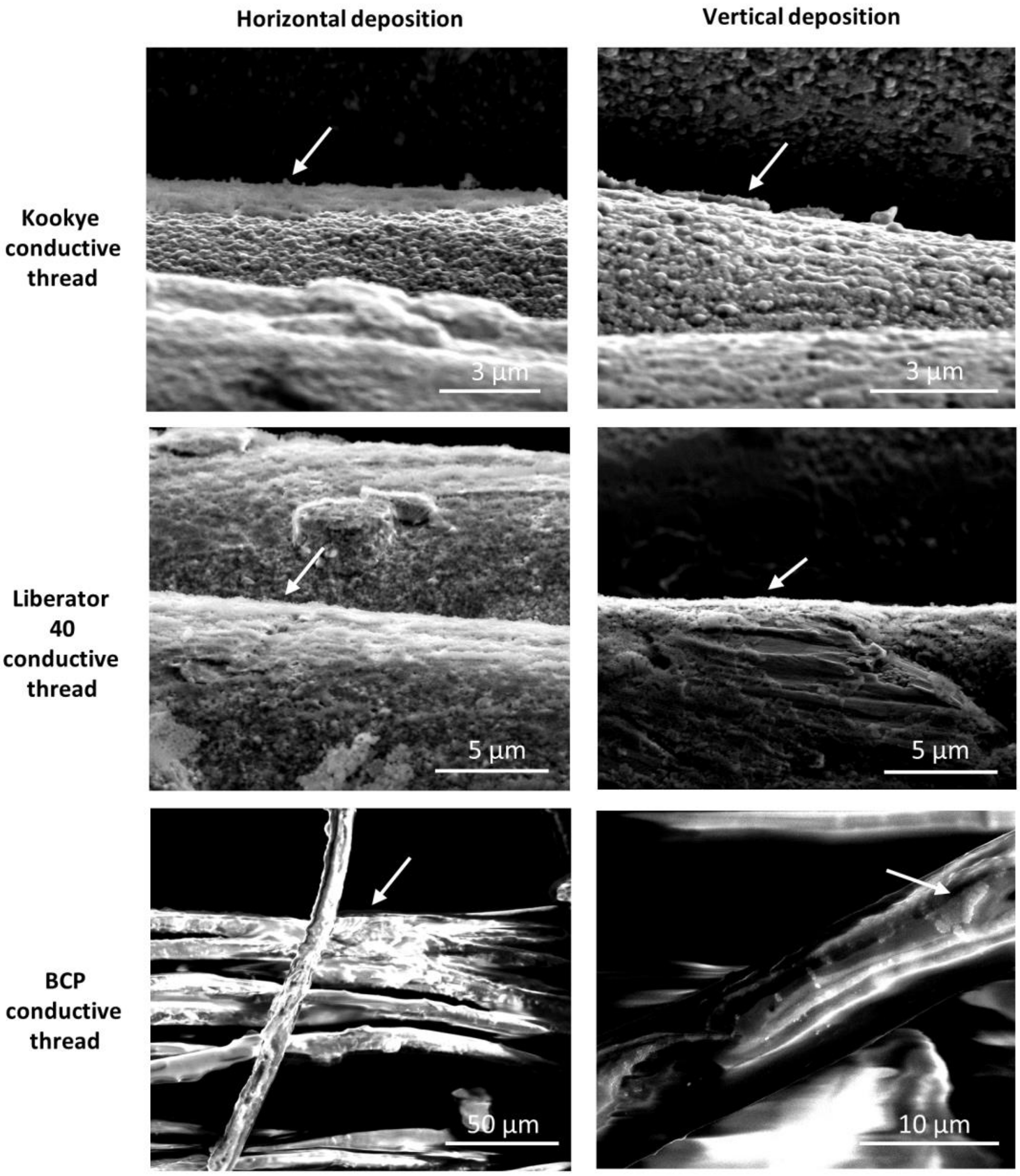

Figure S4. Additional SEM images of the three conductive fibers studied (Kookye conductive thread, Liberator 40 conductive thread, and BCP conductive thread) coated with a LB film of MOF NPs using horizontal (left) and vertical (right) configuration of the fiber holder. The white arrows highlight the MOF NPs deposit.

Table S2. Diameters of different conductive threads measured at five different points in the SEM images of each thread.

Conductive thread type	Diameter (µm)
Kookye	562 ± 24
Liberator 40	310 ± 5
BCP	192 ± 22

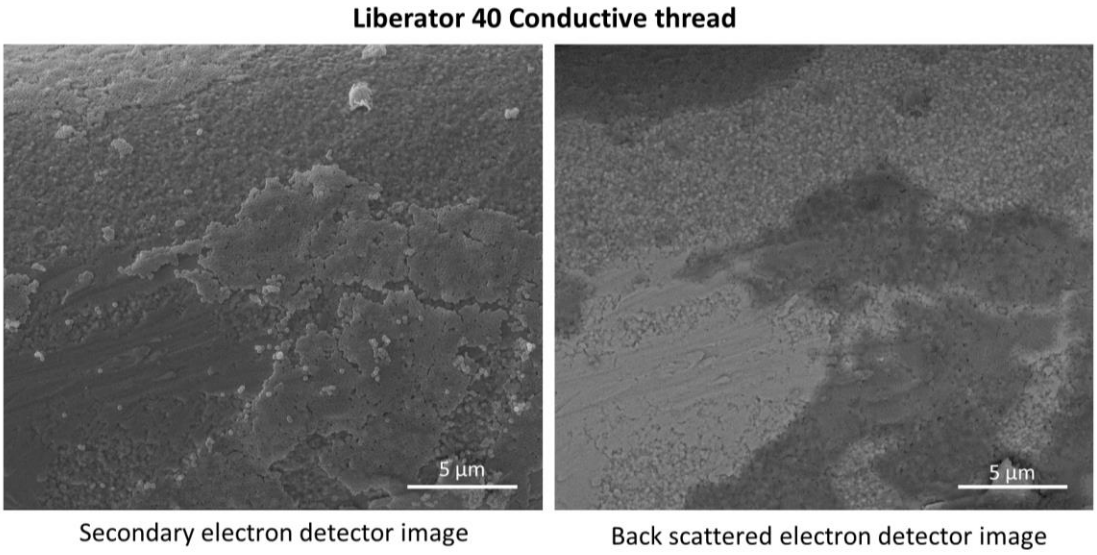

Figure S5. SEM images for a Liberator 40 conductive thread coated with a MIL-96(Al) LB film obtained using either secondary electron (SE) or back-scattered electron (BSE) detectors. MOF covered areas appear as darker zones on the BSE image due to the smaller atomic number.
